# A powerful method for detecting differentially expressed genes from GeneChip arrays that does not require replicates

**DOI:** 10.1186/1471-2105-7-353

**Published:** 2006-07-20

**Authors:** Anne-Mette K Hein, Sylvia Richardson

**Affiliations:** 1 Dept. of Epidemiology and Public Health, Imperial College London, Norfolk Place, London, UK

## Abstract

**Background:**

Studies of differential expression that use Affymetrix GeneChip arrays are often carried out with a limited number of replicates. Reasons for this include financial considerations and limits on the available amount of RNA for sample preparation. In addition, failed hybridizations are not uncommon leading to a further reduction in the number of replicates available for analysis. Most existing methods for studying differential expression rely on the availability of replicates and the demand for alternative methods that require few or no replicates is high.

**Results:**

We describe a statistical procedure for performing differential expression analysis without replicates. The procedure relies on a Bayesian integrated approach (BGX) to the analysis of Affymetrix GeneChips. The BGX method estimates a posterior distribution of expression for each gene and condition, from a simultaneous consideration of the available probe intensities representing the gene in a condition. Importantly, posterior distributions of expression are obtained regardless of the number of replicates available. We exploit these posterior distributions to create ranked gene lists that take into account the estimated expression difference as well as its associated uncertainty. We estimate the proportion of non-differentially expressed genes empirically, allowing an informed choice of cut-off for the ranked gene list, adapting an approach proposed by Efron. We assess the performance of the method, and compare it to those of other methods, on publicly available spike-in data sets, as well as in a proper biological setting.

**Conclusion:**

The method presented is a powerful tool for extracting information on differential expression from GeneChip expression studies with limited or no replicates.

## Background

Affymetrix GeneChips are one of the most widely used commercially available oligonucleotide arrays. They have gained widespread popularity for a number of reasons, among which are their high degree of standardization of the production process and their ability to interrogate tens of thousands of genes simultaneously. They differ from many other array types in that they are one color (and sample) arrays and because each gene is represented by a probe set of pairs of perfect match (PM) and mismatch (MM) probes. Each PM is chosen to match a particular 25 base pair stretch of the sequence encoding the gene, and the full set of PMs is chosen with the aim of uniquely identifying the gene. The accompanying MMs are identical to their PM counterparts except for a complementary base substitution at the middle nucleotide. They are intended to be used to correct for non-specific hybridization. The full set of PMs and MMs for a gene represent the basis for the estimation of the level of expression of the gene.

Most GeneChip based expression studies are carried out with few replicates. There are two major factors behind this: the considerable cost of the GeneChip arrays and limitations on the amount of available RNA. As microarray expression studies are prone to experimental imperfections, failed hybridizations are often encountered resulting in further reduction in the number of replicates available for analysis. If the number of replicates falls below three, most available analysis tools become unsuitable or unapplicable because they rely on the estimation of variances which is difficult in such circumstances. Thus, the development of methods for analyzing experiments with few or no replicates is of high importance.

*B*ayesian *G*ene e*X*pression, BGX [1], is an integrated approach to the analysis of Affymetrix GeneChip arrays. It relies on the formulation of a Bayesian hierarchical model for estimating expression levels from probe level GeneChip data. In the BGX approach background correction, gene expression level estimation and differential expression is performed in an integrated analysis, allowing all uncertainties to be dealt with simultaneously in a coherent statistical framework. Posterior distributions of gene and condition specific BGX expression levels are obtained from a simultaneous consideration of the probe pair intensities in the available probe sets representing the gene. Samples from the posterior distributions are generated using Markov chain Monte Carlo methods. If replicate arrays are available the information in their probe set intensities will be considered jointly in the estimation of expression levels. Replicate arrays, however, are not essential for obtaining the posterior distributions of expression for the genes – these will be obtained from the collection of intensities on the array even when only a single array is available. Samples from the posterior distribution of the differences in expression levels present a basis for inference on differential expression.

In this paper we develop a method for performing differential expression studies from GeneChip experiments without replicates. The procedure exploits the posterior distributions of differences in expression, obtained from a BGX analysis of GeneChip arrays, for creating ranked gene lists. In order to define suitable cut-offs for the list, an estimate of the proportion of differentially expressed genes is obtained by empirically estimating the null distribution of a relevant statistic using an approach similar to that of Efron [2]. The performance of the method is tested on publicly available spike-in data sets and compared to those of other methods, and further evaluated in a biological study.

## Results and discussion

### The BGX model and methodology

Most methods for analyzing GeneChip arrays adopt a stepwise procedure for obtaining a point estimate of expression for each gene on each array. The steps in the procedures typically consist of background correction and normalization followed by summarization (e.g. as in MAS5 [3]) or the fitting of a linear model, often performed on the log-scale background corrected intensities (e.g. as in RMA [4]). Having obtained a point estimate of expression for each gene on each array, studies of differential expression between pairs of conditions are carried out by comparing the collections of point estimates under the conditions using a t-type statistic such as in SAM [5], Cyber-T [6] or Limma [7].

The BGX method differs from these stepwise point estimate approaches in that (1) all steps in the analysis are dealt with simultaneously, (2) gene and condition specific expression levels are estimated from a joint consideration of the available probe set intensities and (3) the outcomes are posterior distributions of expression rather than point estimates. Thus, uncertainties associated with each of the steps are taken into account at all levels of analysis, and the joint uncertainty on the expression level for a gene under a condition is reflected in the shape of the posterior distribution obtained for the level of expression for that gene under that condition.

Explicitly, with *g, j, c *and *r *denoting gene, probe, condition and replicate, respectively, let *S*_*gjcr *_and *H*_*gjcr *_denote gene, probe, condition and replicate specific and non-specific binding (relative to the PM probe) of RNA, and let *φ *∈ (0,1) be a fraction. To further allow for additive array specific errors, e.g. accommodating MMs bigger than PMs, the BGX model hypothesizes:

PM_*gjcr *_~ *N*(*S*_*gjcr *_+ *H*_*gjcr*_, )

MM_*gjcr *_~ *N*(*φS*_*gjcr *_+ *H*_*gjcr*_, ).     (1)

Information on the level of expression of gene *g *under condition *c *is represented by the set of signal parameters representing the gene under this condition: *S*_*gjcr*_, *j *= 1,...,*J*, *r *= 1,..., *R*_*c*_. We assume that these, shifted and logged, come from a gene and condition specific truncated normal distribution. The non-specific hybridization parameters *H*_*gjcr *_reflect characteristics specific to the sample hybridized, leading us to assume array specific truncated normal distributions for these, shifted and logged. Thus,

log(*S*_*gjcr *_+ 1) ~ *TN*(*μ*_*gc*_, ),

log(*H*_*gjcr *_+ 1) ~ *TN*(*λ*_*cr*_, ).     (2)

We will here refer to the *μ*_*gc *_parameters as the BGX expression indices or levels. We assume exchangeability of the gene and condition specific variance parameters,

log() ~ *N*(*a*_*c*_, ),     (3)

with *a*_*c *_and  fixed at values obtained by an Empirical Bayes like approach, thus stabilizing the variance estimation. In all of the above, the distributions are conditional on variables on the right hand side, and independent for all suffices. The model is fully specified by declaring the following, generally weakly informative priors, independent for all suffices: *μ*_*gc *_~ *U*(0, 15), *φ *~ (1, 1), *λ*_*gc *_~ *N*(0, 1000)),  ~ Γ(0.001, 0.001) and (*η*_*gc*_^2^)^2 ^~ Γ(0.001, 0.001)). For a more in-depth discussion of the model we refer to [1].

**Figure 1:**
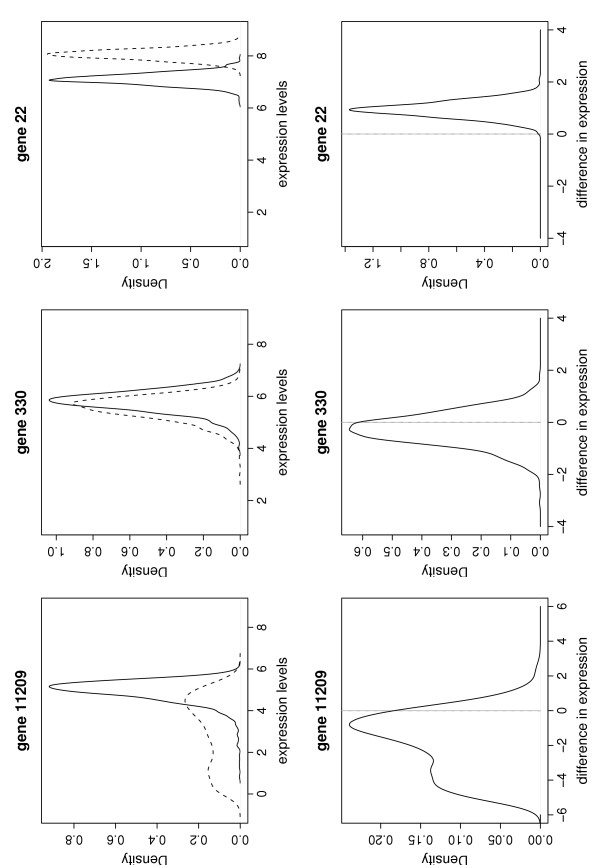
**Posterior distributions of BGX expression levels and their differences**. Kernel density plots of samples of size 1024 from the posterior distributions of the BGX expression indices, *μ*_*g*,1 _(full line) and *μ*_*g*,2 _(broken line) (upper panel), and their differences, *d*_*g *_= *μ*_*g*,1 _- *μ*_*g*,2 _(lower panel), are shown for three genes under two conditions, each represented by a single array.

The BGX model relies on MCMC methods for obtaining samples from the posterior distributions of the parameters. The shapes of the posterior distributions of the BGX gene expression indices, *μ*_*gc*_, are determined by the probe response patterns (see [1]). Thus, a highly consistent probe set response leads to a tight posterior distribution of expression, and a less consistent pattern will result in a flatter, possibly multi-modal, posterior distribution. Examples of posterior expression index distributions, *μ*_*g*,1 _and *μ*_*g*,2_, are given in Figure 1 (upper panel).

The corresponding kernel density plots for the differences in expression indices, *d*_*g *_= *μ*_*g*,1 _- *μ*_*g*,2_, are given in Figure 1 (lower panel). The uncertainties of the expression indices are reflected in the shape of these distributions. For gene 11209 the multi-modality of the posterior distribution of the expression index under condition 2 (*μ*_11209,2_) is reiterated by the multi-modal posterior distribution of the difference in expression. For the other two example genes the posterior distributions of the differences in expression are tight and uni-modal, centered close to zero and around one respectively, indicating similar expression levels for gene 330 and different expression levels for gene 22 under the two conditions considered.

### Addressing differential expression with replicates

A popular approach to conducting differential gene expression studies is to rank the genes according to their degree of evidence for differential expression, and to estimate false discovery rates for different cut-offs on the ranked gene list. This allows the experimenter to obtain a prioritized list of genes to pursue in follow-up studies, with a guidance as to how many genes on the list are expected to be false positives. Such approaches are taken in the implementations of the SAM, Limma and Cyber-T methods. Each of the methods calculate a different modified t-statistic, the modification relating to the standard deviation or variance calculation in the denominator, and genes are ranked on the resulting p-values. In SAM a false discovery rate is estimated based on permuting the original data to get the distribution of (modified) t-statistics under the null-hypothesis of no differential expression. Limma is implemented with the Benjamini and Hochberg method [8] for estimating FDR and calculation of adjusted p-values. Cyber-T adopts the method of Allison et al. [9] for fitting a mixture of Beta-distributions (one of which is the U(0,1) distribution) to the observed p-values, and reports estimated true and false positives along with the posterior probability of differential expression. Thus, all methods make use of point estimates of expression and depend upon replicates being available for estimation of the variance in the modified t-statistics (and in SAM for the permutation).

### Addressing differential expression without replicates

Without replicates the above methods for analysis of differential expression are unapplicable and alternative methods are needed. In this section we describe how the BGX model and methodology may be exploited to obtain such a procedure. We use features of the samples from the posterior distributions collected in the MCMC sampling to produce ranked gene lists. The ranking takes into account the estimated difference in expression level *as well as the associated uncertainty*. We then consider the set of posterior probabilities of expression differences being smaller than zero, *P*(*d*_*g *_< 0), *g *= 1,..., *G*. By comparing their observed distribution to that expected under the null-hypothesis of no differential expression, we obtain an estimate of the number of differentially expressed genes, *G*_*D*_. This allows us to choose the cut-off of the ranked gene list in an informed manner.

The procedures for ranking the genes and for estimating *G*_*D *_in the BGX framework are described in two separate subsections below. The final part of this section contains a comparison of the performance of the BGX based method for performing differential expression analysis from GeneChips without replicates to those of other available methods: the EBarrays method of Kendziorski et al. [10], the Wilcoxon signed rank test for comparison calls of the MicroArraySuite software [3], and the Efron [2] method using the standardized BGX differences (see below) as z-statistics.

#### Ranking genes using BGX

In the BGX framework, the samples from the posterior distributions of the differences *d*_*g *_= *μ*_*g*,1 _- *μ*_*g*,2_, *g *= 1,...,*G*, represent a natural base for inference on differential expression between conditions 1 and 2. These are available irrespectively of the number of replicates for each condition used in the analysis. There are numerous ways in which these posterior distributions can be exploited with the aim of addressing expression differences. Here we study two types of rankings reflecting the potential of the genes as promising candidates for differential expression: (1) ranking on the 'standardized BGX differences', *z*_*g *_= *mean*(*d*_*g*_)/*sd*(*d*_*g*_), where the mean and standard deviation are computed from the posterior sample of *d*_*g *_values, and (2) ranking on the highest percentile, α*, for which the *α*-percent credibility interval for the difference *d*_*g *_does not cover zero. Note that both rankings use the levels of differential expression (the means or the locations of the posterior distributions of the *d*_*g*_*s*) as well as the uncertainty of these (the standard deviation of the posterior sample or the width of the posterior distributions) in the ranking. Without replicates point estimate based methods clearly do not have this ability.

To illustrate ranking (2), consider the posterior distributions of expression index differences in Figure 1 (lower panels). All sampled values from the posterior distribution of *d*_22 _are above zero and α* for gene 22 is indistinguishable from 100%. For gene 330, only very tight credibility intervals exclude zero, and α* for this gene is small (33%). For gene 11209, α* is around 75%.

Rankings of type (1) and (2) differ in their emphasis of the posterior distribution characteristics: type (1) summarizes the full distribution by a traditional t-type statistic (calculated on the posterior sample) and we would expect it to perform well when ranking Gaussian-shape posterior *d*_*g *_distributions (as in Figure 1, genes 330 and 22) and possibly less well in the presence of asymmetric or multi-modal distributions (as in Figure 1, gene 11209). Type (2) rankings use the tails of the posterior *d*_*g *_distributions and should deal equally well with symmetric and asymmetric distributions. However, we rely on a finite sample from the MCMC procedure (we use a sample size of 1024) for approximating the distributions, and the estimation of the tails of the distributions may be fragile.

**Figure 2:**
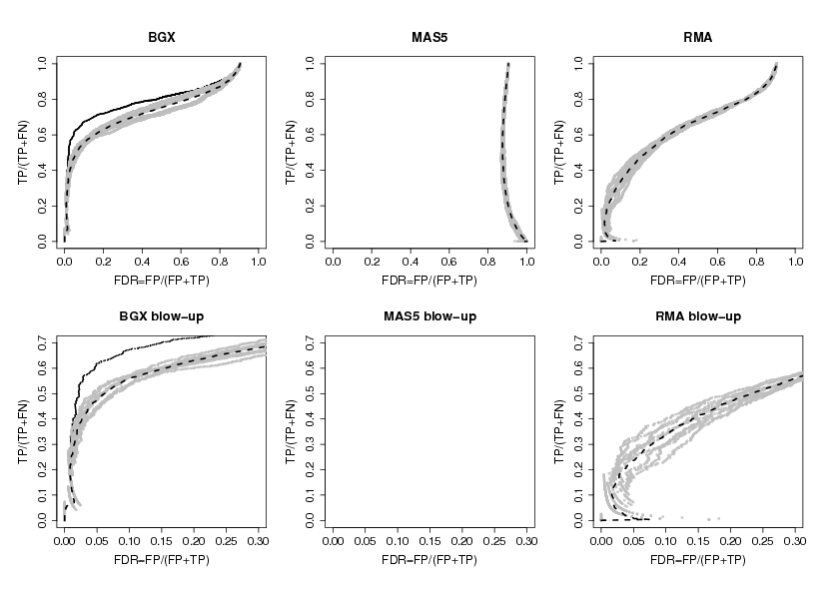
**ROC curves for one versus one comparisons of arrays from the Choe data set obtained with different methods**. A curve is plotted for each pairwise comparison of a single C array to a single S array (9 grey lines) with the average curve superimposed (broken black). For BGX the curve obtained from an analysis that uses all three C replicate arrays against all three S replicate arrays is also shown (full black line). For BGX ranking (1) is used (see text). For MAS5 and RMA genes are ranked on their absolute value of difference in expression. The lower panels are blow-ups of the leftmost parts of the upper panels. TP: true positives, FP: false positives, TN: true negatives, FN: false negatives.

We examined the performance of the two ranking procedures for the BGX method on the results from the nine analyses of pairs of arrays from the Choe data set [11], that consist of a condition C array and a condition S array. On each pair of arrays we ran the BGX model with 2 conditions and 1 replicate per condition, and obtained ranked gene lists using each of the rankings (1) and (2). For the Choe data it is known exactly which genes are differentially expressed (there are 1331 differentially expressed genes out of 14010). This allows the exact numbers of true and false positives and negatives to be calculated, for all possible cut-offs in ranks of the ranked gene lists. The two rankings resulted in almost identical counts, and there is no indication from this analysis that either is to be preferred. ROC curves summarizing the counts obtained with ranking (1) are shown in Figure 2.

The performance of the BGX-based rankings from the single replicate comparisons in the Choe data set is remarkable: a quarter of the 1331 truly differentially expressed genes are included in gene lists with realized false discovery rates of 0.02 (gene list length approximately 500). By extending the gene list to 700 genes (5% of the total number) the proportion of truly differentially expressed genes detected is increased to 50% and the realized false discovery rate thus about 6%. Gene lists of lengths 1300 include 70% of the truly differentially expressed genes and have observed false discovery rates of approximately 30%. Furthermore, the curves for the nine different analyses of pairs of arrays are highly similar, indicating a stable performance. For comparison, ROC curves obtained for the same pairwise analyses using MAS5 and RMA are also given in Figure 2. For these analyses, genes were ranked on the absolute values of the differences in expression measures (obtained with RMA or MAS5) between conditions. With just one replicate array per condition, uncertainty of the estimates of expression is not accounted for by these methods, and they both do less well than the BGX-based method. Due to the high variability of MAS5, this method performs particularly poorly in the one-versus-one array rankings.

**Figure 3:**
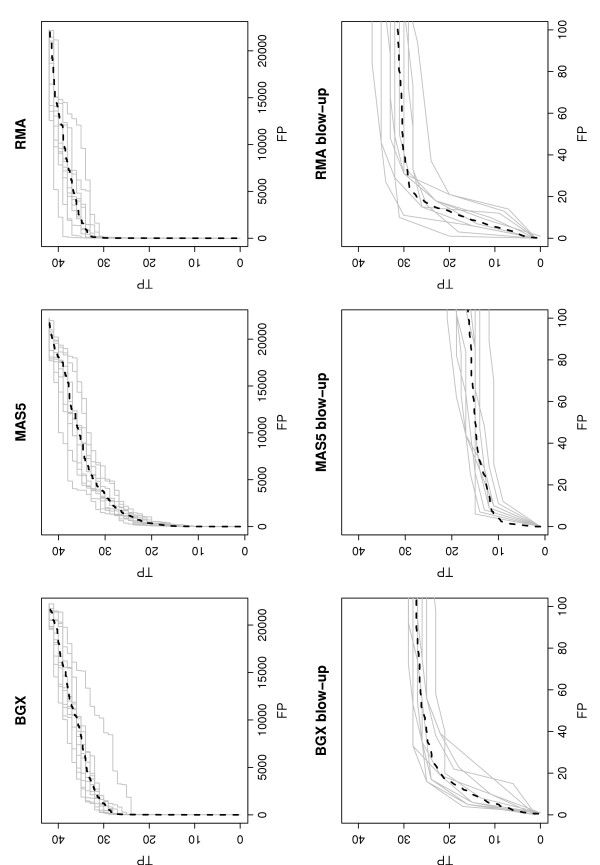
**ROC curves for one versus one comparisons of arrays from the AffyU133A data set obtained with different methods**. (see legend of Figure2).

To put the above performances into perspective the ROC curve obtained for the same method of ranking but using results from a BGX analysis that uses all the available arrays (two conditions, three replicates each) are also shown in Figure 2. For this analysis the number of truly differentially expressed genes included in the lists are increased by 25, 10 and 5% respectively to 50, 60 and 75% for the levels of realized false discovery rates of 0.02, 0.06 and 0.3. Thus, as expected, gene ranking is improved when all replicates are used. However, the proportion of the information contained in the full data set, that may be extracted from the single replicate analyses array is considerable. We note that the performance of the BGX multiple array model on the full Choe data set is among the methods found to perform best (see [10], Figure 7). Results from a similar analysis on the much less extensive AffyU133A spike-in data set [12], are shown in Figure 3. As only 42 of the 22300 genes represented in this data set are differentially expressed, we plot absolute rather than fractional values of true and false positives. For this data set the ranking of the top genes produced by the three methods are similar. For gene lists longer than 15, BGX and RMA outperform MAS5, and RMA performs better than BGX for lengths above 35. Thus, the relative performances of the methods differ for the Choe and the AffyU133A data sets. This is most likely due to the different levels of noise in the two data sets. With only 42 spike-in genes in the AffyU133A data set arrays from two different conditions are almost like technical replicates. In the Choe data set, the 1331 genes spiked-in at varying concentrations at small to moderate fold changes result in a noticeable, and more biologically realistic, level of noise between the two conditions compared. Thus, for the Choe data set, noise has an impact and accounting for this, as is done with the BGX based ranking, is essential, whereas for the AffyU133A data set, the impact of noise is negligible and the importance of taking this into account is outweighed by that of reproducibility of point measures for (almost) technically replicate arrays.

#### Estimation of the proportion of differentially expressed genes

Having obtained a ranked list of genes the next question is whether we can choose a suitable cut-off. Depending on the downstream goal, we may wish to arrive at a (long) list that has a good chance of containing most or all of the "interesting" genes (meaning those that are differentially expressed), accepting that false positives will also be included, or we may prefer to end up with a (short) list of genes most of which would appear to be promising candidates for differential expression, expecting very few false positives to be included. To guide the choice of cut-off, it is useful to obtain an estimate of the proportion of differentially expressed genes. To do this, we estimate empirically the distribution of a relevant statistic under the null hypothesis, which in turn allows for the quantification of the proportion of non-null behaving genes, following the idea of Efron [2].

We consider, for each gene *g*, the posterior distribution of the difference in expression, *d*_*g *_= *μ*_*g*,1 _- *μ*_*g*,2_, obtained from the BGX analysis. Under the null-hypothesis of no differential expression this posterior distribution should be centered on zero. Rephrasing in terms of the posterior *P*(*d*_*g *_< 0) probability, under the null we expect this to be 0.5. Considering the posterior *P*(*d*_*g *_< 0) probabilities for the full set of genes analyzed, under the null hypothesis of no differential expression there should be decreasingly less support for values away from 0.5 towards 0 and 1. Thus, with no differentially expressed genes a histogram of the *P*(*d*_*g *_< 0) probabilities should be uni-modal with mode of approximately 0.5, and have smoothly decreasing tails. The width of the central component of the histogram will depend on the posterior distributions of the *d*_*g*_'s: the less clean the information on the expression levels (e.g. the more noisy the data), the less tight the *d*_*g *_distributions, and thus the flatter the histogram of *P*(*d*_*g *_< 0) values. (We have phrased the above in terms of the *P*(*d*_*g *_< 0) values but could of course equally well have phrased them in terms of the *P*(*d*_*g *_> 0) values).

**Figure 4:**
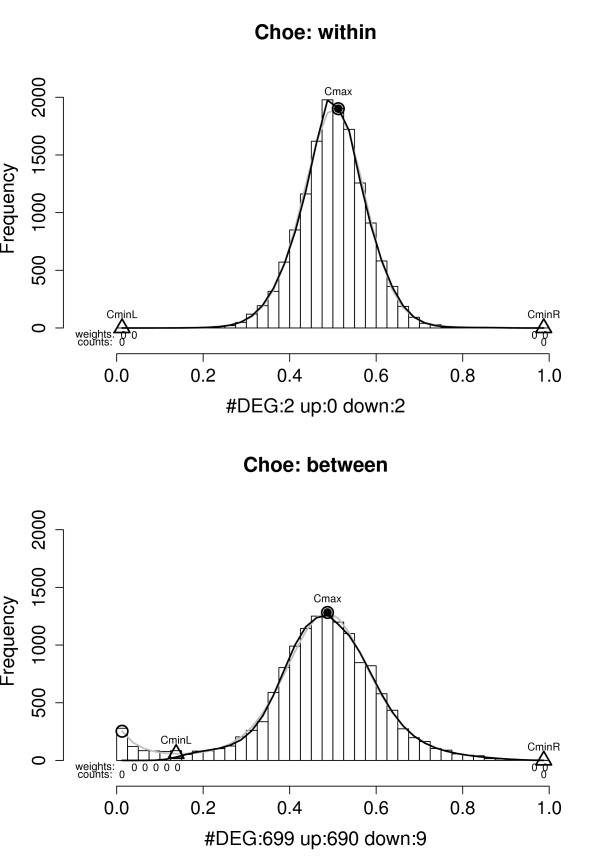
**Example histograms of the sets of *P*(*d*_*g *_< 0) values for within and between condition single replicate comparisons – Choe data set**. Results shown are for analyses of condition C replicate 1 against condition C replicate 2 (upper panel) and condition C replicate 1 against condition S replicate 2 (lower panel). Grey curve: *f*, black curve: *f*_0_, grey circles: local maxima, grey triangles:  and , black point in grey circle:  (see text). DEG: estimated number of differentially expressed genes, ."weights": a 0 indicates that the category is given zero weight when fitting *f*_0_. "counts": a 0 indicates that the category is fixed to have count zero when fitting *f*_0_. "up" and "down" denote the estimated number of over- and under expressed genes,  and . The upper histogram exhibits the shape expected under the null-hypothesis of no differentially expressed genes (the grey and black curves overlap), the lower indicates the presence of a subset of over-expressed genes.

Deviations in a histogram of *P*(*d*_*g *_< 0) values from the expected shape under the null hypothesis of no differential expression indicate the presence of differentially expressed genes: excess of *P*(*d*_*g *_< 0) values near zero and one indicate over-expressed and under-expressed genes in condition 1 relative to condition 2, respectively. To quantify the number of non-null genes, we adopt an approach similar to that of Efron [2]. We fit a polynomial, *f*, to the histogram counts by Poisson regression and use the central part of the histogram to estimate the null component, while excess area in the tails will represent differentially expressed genes. To be precise, we use the following procedure (see Figure 4 for illustration): We identify the inner global max and left and right most minima on the fitted curve *f*, and refer to the histogram categories of these points as *c*_*max*_,  and  respectively. We use the central part of the histogram (that between categories  and ) to obtain the distribution of the *P*(*d*_*g *_< 0) values under the null: we fit two new curves,  and , to the parts of the histogram left and right of category *c*_*max*_, respectively, using the same fitting procedure as for the curve *f *but fixing the histogram counts of the outermost categories *c*_1 _and *c*_*K *_to zero (with *K *denoting the total number of categories in the histogram), and giving zero weight to categories *c*_2_,...,  and ,..,*c*_*k*-1 _respectively. An estimate of the number of genes under the null is obtained by summing the fitted values of the empirical null distribution *f*_0_,



Denoting the total number of genes by *G*, the estimated proportion of differentially expressed genes is



Estimated numbers of over- and under-expressed genes,  and  (in condition 1 relative to 2), are obtained by quantifying the excess genes in either tail of the histogram relative to those expected under the null. Denoting the histogram count in category *c*_*i *_by *h*(*c*_*i*_) we set





**Figure 5:**
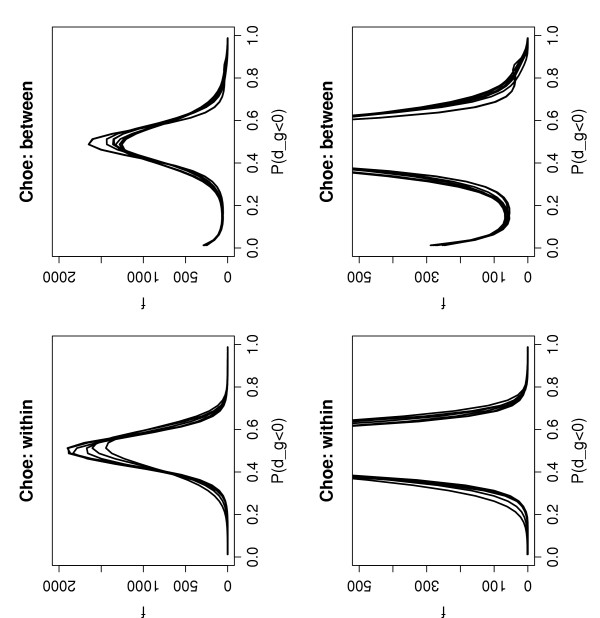
**Plots of the curves (*f*, see text) fitted to the histogram counts of *P*(*d*_*g *_< 0) values for pairwise within and between condition comparisons – Choe Data set**. A curve is plotted for each pair of arrays in the Choe data set (left: within condition comparisons, right: between condition comparisons). The plots show the expected shape of uni-modality and smoothly decreasing tails under the null (left) and deviations from this pattern, largely in terms of an excess of over-expressed genes in the right-hand tails, for the between condition comparisons (right).

We applied the above method to the BGX analyses of the pairs ofarrays from the Choe and AffyU133A data sets. An example of a histogram of *P*(*d*_g _< 0) values from a *within condition *comparison of two arrays from the Choe data set is given in Figure 4, upper panel. The full set of histograms for *within condition *analyses of pairs of Choe arrays are summarized in Figure 5, left, in terms of plots of the curves, *f*, fitted to the histogram counts by Poisson regression. For within condition comparisons the arrays are replicates, so there should be no differentially expressed genes, and the plots indeed exhibit the shape expected under the null hypothesis: they have a central mode near 0.5 and smoothly decreasing tails. They thus confirm our expectations and indicate that the method works well under the null. Figure 4, lower panel, and Figure 5, right panel, display the equivalent plots for pairwise *between condition *analyses of the Choe data set arrays. The histograms for these analyses exhibit a clear deviation from the shape expected under the null in terms of an excess of small *P*(*d*_*g *_< 0) values near zero indicating the presence of over-expressed genes. The right-hand tails of the histograms decrease smoothly and there is no indication of under-expressed genes. This is exactly the pattern that should emerge for the Choe between condition comparisons: all differentially spiked-in genes have higher concentration under condition S (our condition 1) than under condition C (our condition 2). The estimates of the numbers of differentially expressed genes obtained for the Choe data are summarized in Table 1. For the within-condition analyses the estimates of the proportions of differentially expressed genes are near zero, indicating high specificity of the method. For the between condition analyses, the number of differentially expressed genes is estimated to be approximately 700. Of the genes declared differentially expressed approximately 95% are true positives, demonstrating the methods high positive predictive value.

**Figure 6:**
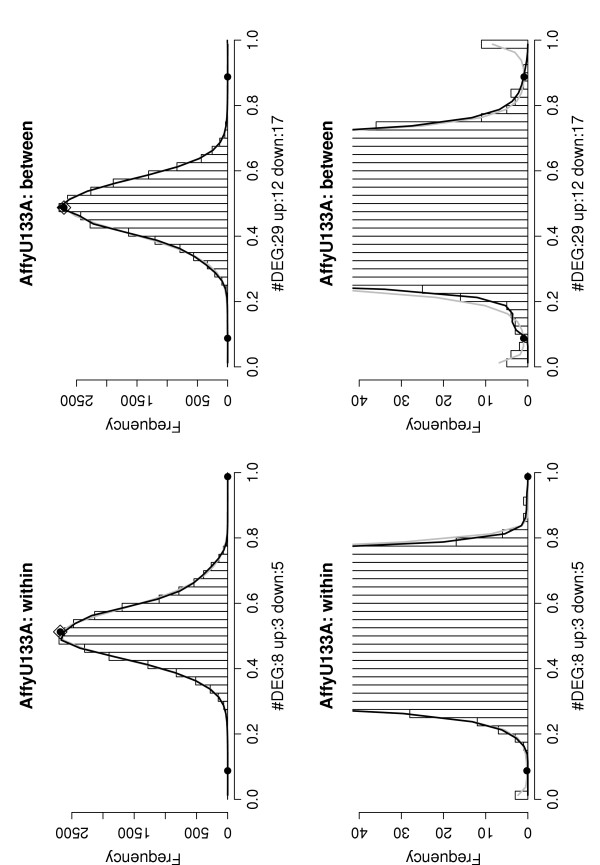
**Example histograms of the sets of *P*(*d*_*g *_< 0) values for within and between condition single replicate comparisons – AffyU133A data set**. Results shown are for analyses of two arrays from the same experiment (Experiment 3, replicates 1 and 2, left) and two arrays from different experiments (replicate 1 from Experiment 3 and 4, right). Lower panels are blow-ups of the lower part of the upper panels. Notation is as in Figure 4. The histogram on the left exhibits the shape expected under the null-hypothesis of no differentially expressed genes (the grey and black curves overlap), that to the right indicates the presence of small subsets of over- and under-expressed genes.

A similar analysis on the AffyU133A data is summarized in Figure 6, supplementary Figure 1 [see Additional file 1] and Table 1. For this data set the histograms of the *P*(*d*_*g *_< 0) values obtained for the replicate array comparisons and the between experiment comparisons are more similar. With the very few spike-in genes, and thus little difference between the within and between experiment analyses in this data set, this is expected. Also note that the central component is tighter for the AffyU133A data set than for the Choe data set, reflecting the lower level of noise. Focusing on the tails of the histograms for the analysis of arrays from different experiments (right), there is a clear deviation from the shape expected under the null, in terms of an excess of *P*(*d*_*g *_< 0) values near 0 as well as 1, indicating the presence of both over- and under-expressed genes. The estimated numbers of differentially expressed genes in the between experiment analyses are around 30, and only a couple for the within experiment analyses. Of the genes declared differentially expressed in the between condition comparisons approximately half are true positives. Thus, in spite of the relatively low fold changes of 2 in this data set, the method retains good sensitivity and high specificity.

#### Comparison to other methods

Few methods are available for performing differential expression analysis from GeneChip arrays in the absence of replicates. Here we compare the performance of the BGX based method to those of three other methods that may be applied when only a single replicate is available: the EBarrays method of Kendziorski et al. [10] and the Affymetrix MAS comparison calls [3]. The MAS comparison calls are based on the Wilcoxon signed rank test applied, for each gene, to the sets of PM-MM values on the two arrays to be compared. The Wilcoxon signed rank test is available in R. We rank the genes on their p-values and use as cut-off the recommended value of 0.0025 to declare the genes as differentially expressed ([3]). The EBarrays method (available from Bioconductor, [13]) implements the empirical Bayes Gamma-Gamma or lognormal-normal mixture models, originally developed for two-colour cDNA arrays, but equally applicable to analysis of differential expression between GeneChip arrays. We use the lognormal-normal model, as generally recommended for GeneChip data, and apply the method to both the RMA expression values (transformed to the original scale) and the MAS5 values. The method estimates the proportion of non differentially expressed genes, *p*_0_, and for each gene, their posterior probability of belonging to the non-null component. We rank the genes following decreasing values of this probability and compute the rank cut-off by multiplying *p*_0 _by the total number of genes analysed. As an additional comparison, we show results for the Efron (2004) method applied to the standardised BGX differences *z*_*g *_= *mean*(*d*_*g*_)/*sd*(*d*_*g*_), *g *= 1,...,*G*.

The method fits a spline to histogram counts of the *z*_*g *_values, using Poisson regression. The null distribution is then empirically estimated from the observed distribution by a first and second moment fitting of a normal distribution to the central component, and used to produce an estimate of *p*_0_. The genes are ranked on their absolute *z*_*g *_values and a rank cut-off is estimated as for the EBarrays method above. Estimated numbers of differentially expressed genes obtained with the various methods are given in Table 1, along with numbers and proportions of true positives among those declared differentially expressed. The specificity and positive predictive value is highest for the BGX based method. Among the other methods, the EBarrays method with RMA expression measures performs best. At the opposite extreme is the EBarrays method with MAS5 expression values: this combination results in very poor specificity and lowest positive predictive value of the methods tried. The poor performance is likely to be caused by the high variability and strong mean variance relationship of the MAS5 measure, which in combination with the assumption of a common variance for the genes and constant coefficient of variation of the EBarrays model is detrimental. In contrast, the RMA method has low, stable, variance across the genes, and thus conforms well to the assumptions of the EBarrays method. Efron's method applied to the standardised BGX differences has relatively high positive predictive value (between that of the BGX method and the EBarrays with RMA). However, as seen in the large number of wrongly declared differentially expressed genes in the within condition comparisons, this is achieved at the expense of high numbers of false positives. This shows that the approximation of the null component of the histogram of standardised BGX differences by a Gaussian distribution in the Efron method is not entirely appropriate and that a more flexible, non-parametric fit is indeed needed. Finally, the performance of the Wilcoxon signed rank test is relatively poor on both positive predictive value and specificity.

### A biological example

The applications of the method on the spike-in data set analyses illustrate the performance of the method for detecting and estimating the proportion of genes that have been spiked into a common pool of RNA at different concentrations. This is a somewhat stylized situation and, in practice, what we are interested in is the performance of the proposed method in analyses of realistic biological data sets.

The virtue of the spike-in data sets is that we know exactly which genes are differentially expressed. The problem with testing the method on a biological data set is that there are no biological data sets which possess this feature. In the absence of such a data set we consider instead a biological data set for which we will regard part of the truth as known: the Lin data set [14]. Lin et al. conducted a time course experiment on mice, with the aim of identifying hair cycle-associated genes. In mice, the first two hair growth cycles are synchronous, but after these, hair growth proceeds asynchronously. In the Lin et al. study, the expression of genes in mouse back skin was measured at 5 time points taken during the first hair-growth cycle and at three time points after the second hair-growth cycle. The authors hypothesized that genes related to hair-growth cycle should exhibit increased replicate variance from the synchronous to the asynchronous phase, and identified 2289 genes for which the replicate variance was significantly increased. To validate their findings they went through a literature search and compiled a list of 89 genes that have been shown to be hair cycle-dependent by using other methods (e.g. RT-PCR). Of the compiled list of 89 genes, 72 were among the 2289 identified by Lin et al's increased replicate variance based method on the GeneChip data, thus verifying that hair-cycle associated genes were found by their method. The time-course profiles of the 2289 genes were subsequently clustered, the clusters studied and found to relate to distinct genetic pathways.

We will use the literature-based compiled list of 89 hair cycle-associated genes as a starting point for making a list of what we will assume to be a subset of the list of "truly differentially expressed genes" between two select time points in the Lin data set. Note that a hair-cycle associated gene need not be differentially expressed at any pair of the time points studied. An examination of the time course cluster profiles indicates that a sizeable subset of the hair-cycle associated genes may have different expression levels at time points 14d and 23d (both within the first hair-cycle), and we choose to consider arrays from these two time points. Of the 89 genes in the literature compiled list, only the 72, that were included in the list of the 2289 genes were clustered, and we thus only have information on the expression levels at the two considered time points for these 72 genes. The cluster membership of the 72 genes suggest that 11 belong to clusters with similar levels of expression at time points 14d and 23d. Thus, we are left with a list of 61 genes, from the initial list of 89, that: (1) have been shown to be hair-cycle associated with other methods such as RT-PCR, and that, (2) we would expect to have different levels of expression at time points 14d and 23d (as judged from inspection of the clusters to which they belong). Importantly, there may well be many other genes on the arrays, apart from those associated to the hair growth cycle associated genes, that are differentially expressed at the two time points considered. Thus our assumption is that *the list of 61 is a subset of the list (of unknown length) of "truly differentially expressed genes at the two time points"*. We take as a criterion for success the ability of the method to detect as differentially expressed these 61 genes – allowing that more genes than 61 may well be differentially expressed.

**Figure 7:**
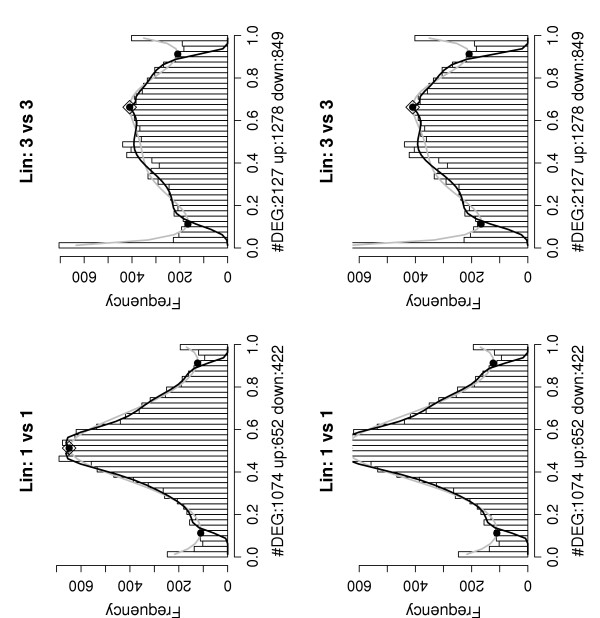
**Histograms of the *P*(*d*_*g *_< 0) values – Lin data set**. Results shown are for the two arrays from mouse 1 at time points 14d and 23d (left) and from mice 1, 2 and 3 at time points 14d and 23d (right). Both analyses show an excess of *P*(*d*_*g *_< 0) values in the tails indicating the presence of both over- and under expressed genes.

Figure 7, left, shows a histogram of the *P*(*d*_*g *_< 0) values for the analysis of the two arrays that measure the expression levels in mouse 1 at the time points 14d and 23d. As expected, the central mode of the histogram is considerably wider than those for the Choe and AffyU133A data sets, reflecting the larger levels of noise in this truly biological sample comparison. In spite of this, an excess of genes in both tails of the histogram is clearly visible and we arrive at estimates of approximately 650 over-expressed and 420 under expressed genes or approximately 7.5%. An examination of the identity of the top 1000 genes reveal that they include 42 of the 61 genes from our assumed sublist of "truly differentially expressed genes". Of the 42, 38 are ranked in the top 500. Thus, a large proportion of the "known" truly differentially expressed genes are thus found by the differential expression analysis with just a single replicate for each condition. As a further examination of the performance of our method, we analyzed all the available arrays in the Lin data set at time points 14d and 23d, using the BGX model with two conditions of three replicates each. Figure 7, right, shows the histogram of *P*(*d*_*g *_< 0) values for this analysis. The central component of the histogram of *P*(*d*_*g *_< 0) values is much flatter for this analysis than for the one mouse at time points 14d and 23d analysis. This is expected: the histograms to the left relate to a comparison of expression levels at two time points *for the same mouse*, where as the histograms to the right are for a comparison of expression levels at two time points between three sets of mice. Although the three mice studied at the two time points are the same, this is not taken into account in the analysis, and the comparison is merely of average time-point effects. Thus the *d*_*g *_distributions in the three against three mice analysis include variability among mice within each time point. Nevertheless, there is still a clear indication from the histogram of an excess number of over- as well as under expressed genes, the number estimated to be approximately 2130 (15% of the genes monitored). Examining the identity of the genes we find that the 2000 highest ranked genes include the 42 genes from the list of 61 found in the without replicates analysis of the arrays for mouse 1 (39 of these are in the top 500), and an additional 4. We take this as a further validation of the procedure's ability to extract valuable information on differential expression from GeneChip analysis in biological studies without replicates.

## Conclusion

We described a method for performing differential expression analysis from GeneChip arrays which does not require replicates. In the method the posterior distributions of the expression differences are used to obtain ranked gene lists and to estimate the proportion of differentially expressed genes. We investigated the performance of the method for analysis of one single array against another and found the method to perform well on controlled data sets as well as in a biological setting. The method is not limited to analysis of one single array against another, but is indeed applicable to analyses of any one number of replicates against any other number.

The method for estimating the proportion of differentially expressed genes relies on the empirical estimation of the null distribution of the *P*(*d*_*g *_< 0) values. For this, we modified the method of Efron [2] in two ways. Efron's method operates on standardised differences and fits a normal to the central part of the observed distribution of these values. We found that the normal assumption for the null component of the standardised BGX differences did not produce convincing results. Instead, we chose to use the natural quantity: *P*(*d*_*g *_< 0) that compares the expression difference to its value under the null, and to exploit its expected shape under the null. The values of *P*(*d*_*g *_< 0) are bounded by 0 and 1, and there is no reason to expect that the symmetric smoothly central component of their distribution should follow any particular parametric shape. Fitting a mixture of normal distributions with a variable number of components as in Richardson and Green [15] results in the central (that is, null) component being fitted by a number of normals with means near 0.5, and different variances. This makes characterising which components of the mixture make up the null difficult. In the procedure that we describe we have opted instead for simplicity and attempting to stay close to the empirical distribution by using a spline fit, rather than fitting a particular parametric form.

A different approach to differential expression analysis in the BGX framework would be add an additional hierarchical level to our BGX model and to define a mixture prior for the expression levels rather than a flat prior. This approach has be taken by a number of authors, for example Kendiorski et al. [10] and Gottardo et al. [16], working from (summary) expression level data, rather than probe level intensities. Such an extended BGX model would be statistically and theoretically appealing, but also computationally demanding, and will be explored in future work.

## Methods

### Data sets

We use arrays from three data sets: the Choe data set, the AffyU133A data set and the Lin data set. The data sets are described below:

#### The Choe data set

We use the full Choe data set [10] which is a spike-in study, consisting of 6 arrays, representing two conditions, each with three replicates. The arrays are Drosgenomel GeneChips with 14010 probe sets. The samples hybridized to the arrays consist of mRNA for 3860 genes at known concentrations, with 1331 of the 3860 genes having different concentrations under the two conditions. Fold changes range from 1.2 to 4, and, when different, concentrations are highest on condition S arrays. Thus of the 14010 genes represented on the arrays, 10150 should not be expressed, 3860 should be expressed, and 1331 of the 3860 expressed genes should be differentially expressed under the two conditions. The Choe data set represents more realistic levels of between-condition noise than most other available spike-in data set, because the proportion of genes with differing concentrations is substantial (approximately 10%). As for other spike-in data sets, the within condition noise is low due to the material on the arrays being technically replicate. For the Choe data set there are 9 between condition analyses of a single C to a single S array, and 6 within condition analyses of either two condition C or two condition S arrays.

Because all genes are spiked up in concentration on the S arrays relative to the C arrays in this data set particular care must be taken in the normalization. In these analyses we have replaced the normalization employed by default by each of the methods by the following normalizations: for the BGX, RMA and MAS5 methods, expression measures were first calculated without normalization. The obtained expression levels were subsequently normalized using a flexible loess (span 0.1) normalization calculated from the subset of non changing genes only. For the Wilcoxon signed rank analysis which is carried out at the probe level, the MAS5 trimmed mean scaling was employed, using the probes for the non-differentially expressed genes only.

#### The AffyU133A data set

We use replicates 1 and 2 from each of the 14 experiments in the Affymetrix Latin Square data set [12]. All arrays in this experiment have technically replicate samples of RNA hybridized, except for the spiking-in material of 42 genes at known concentrations, differing between experiments. We analyze the pairs of replicates for each experiment, *k *= 1,...,13, as well as the pairs of arrays from subsequently numbered experiments, *k *and *k *+ 1, for *k *= 1,...,13. The fold changes in the spike-in concentrations in these between experiment comparisons are 2 for 39 of the 42 genes, and ∞ for the remaining 3. As the AffyU133A arrays have 22300 probe sets, a very small proportion is differentially expressed (42/22300, or 0.5 %). Due to the technically replicate material used for all arrays, and the low number of spike-in genes there should be very little noise between as well as within conditions for this data set.

#### The Lin data set

Lin et al [14] conducted a time course experiment on mice, with the aim of identifying hair cycle-associated genes, using MGU74Av2 GeneChips. These arrays contain 14010 genes. We use two arrays from mouse 1 for time points 14d and 23d (arrays GSM34315 and GSM34322) for analysis without replicates. All six mouse arrays at the two time points 14d and 23d are used in the two conditions with three replicates each analysis.

## Authors' contributions

The authors contributed equally to the presented work.

## Supplementary Material

Additional File 1 **Supplementary Figure 1 – Plots of the curves (*f*, see text) fitted to the histogram counts of *P*(*d*_*g *_< 0) values for pairwise within and between condition comparisons – AffyU133A Data set**. A curve is plotted for each pair of arrays in the AffyU133A data set (left: within condition comparisons, right: between condition comparisons). The plots show the expected shape of uni-modality and smoothly decreasing tails under the null (left) and deviations from this under this pattern, in terms of excess values near 0 and 1, for the between condition comparisons (right). Click here for file

## Figures and Tables

**Table 1:** estimated numbers of differentially expressed genes. Estimated numbers of differentially expressed genes (numberDEG) for between (left part of table) and within (right part of table) condition analyses. For the between condition analyses the numbers of true positives (number TP) and proportions of true positives among those declared differentially expressed (proportion TP among DE) are also given (middle part of table). The upper part of the table shows result for the Choe data set, the lower for that of the Affy data set. Each row gives results for a particular method on the given data set. Shown are, for each data set and method the mean (in bold) and the minimum and maximum values (in parentheses) obtained over all the analyses of pairs of arrays performed. The BGX method has the highest positive predictive value and sensitivity.

	between			within
	number DEG	number TP	proportion TP among DE	number DEG

Choe data set:				
BGX (*P*(*d*_*g *_< 0))	**705 **(507,882)	**674 **(499,826)	**0.961 **(0.937,0.984)	**4 **(0,8)
BGX (Efron z-stat)	**1161 **(786,1448)	**862 **(738,949)	**0.757 **(0.647,0.939)	**512 **(185,830)
EBarrays(RMA)	**773 **(519,1006)	**570 **(448,686)	**0.754 **(0.574,0.863)	**13 **(0,80)
EBarrays(MAS5)	**347 **(240,443)	**2 **(1,4)	**0.008 **(0.002,0.013)	**742 **(266,1141)
Wilcoxon	**2940 **(2827,3019)	**806 **(755,840)	**0.274 **(0.267,0.278)	**53 **(29,242)

Affy data set:				
BGX (*P*(*d*_*g *_< 0))	**28 **(4,57)	**13 **(6,25)	**0.510 **(0.333,0.800)	**2 **(0,14)
BGX (Efron z-stat)	**574 **(318,894)	**26 **(23,28)	**0.041 **(0.029,0.075)	**736** (365, 942)
EBarrays(RMA)	**69 **(40,100)	**28 **(19,35)	**0.423 **(0.310,0.542)	**7 **(0,22)
EBarrays(MAS5)	**2831 **(2610,3134)	**12 **(5,20)	**0.004 **(0.002,0.007)	**2888 **(2592,3115)
Wilcoxon	**114 **(75,167)	**4 **(3,6)	**0.031 **(0.018,0.057)	**102 **(71,186)
